# Pyranocoumarins from Root Extracts of *Peucedanum praeruptorum* Dunn with Multidrug Resistance Reversal and Anti-Inflammatory Activities

**DOI:** 10.3390/molecules201219738

**Published:** 2015-11-25

**Authors:** Jun Lee, You Jin Lee, Jinhee Kim, Ok-Sun Bang

**Affiliations:** 1 KM Convergence Research Division, Korea Institute of Oriental Medicine, Daejeon 34054, Korea; junlee@kiom.re.kr (J.L.); ojung8384@kiom.re.kr (Y.J.L.); jinheekim@kiom.re.kr (J.K.); 2 Korean Medicine Life Science, University of Science & Technology, Daejeon 34054, Korea

**Keywords:** *Peucedanum praeruptorum*, Umbelliferae, pyranocoumarin, multidrug resistance, anti-inflammation

## Abstract

In the search for novel herbal-based anticancer agents, we isolated a new angular-type pyranocoumarin, (+)-*cis*-(3′*S*,4′*S*)-3′-angeloyl-4′-tigloylkhellactone (**1**) along with 12 pyranocoumarins (**2**–**13**), two furanocoumarins (**14**, **15**), and a polyacetylene (**16**) were isolated from the roots of *Peucedanum praeruptorum* using chromatographic separation methods. The structures of the compounds were determined using spectroscopic analysis with nuclear magnetic resonance (NMR) and high-resolution-electrospray ionization-mass spectrometry (HR-ESI-MS). The multidrug-resistance (MDR) reversal and anti-inflammatory effects of all the isolated compounds were evaluated in human sarcoma MES-SA/Dx5 and lipopolysaccharide (LPS)-induced RAW 264.7 cells. Among the 16 tested compounds, two (**2** and **16**) downregulated nitric oxide (NO) production and five (**1**, **7**, **8**, **11**, and **13**) inhibited the efflux of drugs by MDR protein, indicating the reversal of MDR. Therefore, these compounds may be potential candidates for the development of effective agents against MDR forms of cancer.

## 1. Introduction

The dried roots of *Peucedanum praeruptorum* Dunn (Umbelliferae) are a well-known traditional Chinese medicine, Bai-hua Qian-hu that is officially listed in the Chinese Pharmacopeia and has been used as an antipyretic, antitussive, and in the treatment of allergic asthma [1,2]. Phytochemical and pharmacological studies showed that angular-type pyranocoumarins are the major constituents of this plant [3,4,5,6,7,8,9], and these compounds have various beneficial effects such as anti-inflammatory [10,11,12], antiasthma [13], chemopreventive [14], smooth muscle relaxant [15], neuroprotective [16], and anti-osteoclastogenic properties [17].

As a part of the ongoing projects for the discovery of new anticancer drugs from traditional herbal medicines, chromatographic separation of a 70% ethanol (EtOH) extract of the roots of *P. praeruptorum* led to the isolation of a new angular-type pyranocoumarin (**1**), along with 15 compounds: 12 pyranocoumarins (**2**–**13**), two furanocoumarins (**14**, **15**), and a polyacetylene (**16**). The structures of the isolates were determined spectroscopically using one-dimensional (1D)- and two-dimensional (2D)-nuclear magnetic resonance (NMR) analysis. All the compounds (**1**–**16**) were evaluated for multidrug resistance (MDR) reversal and anti-inflammatory activity against multidrug resistant MES-SA/Dx5 cancer and lipopolysaccharide (LPS)-stimulated RAW 264.7 cells. Here, we report the isolation, structural elucidation, and biological activities of these compounds isolated from the roots of *P. praeruptorum*.

## 2. Results and Discussion

**Figure 1:**
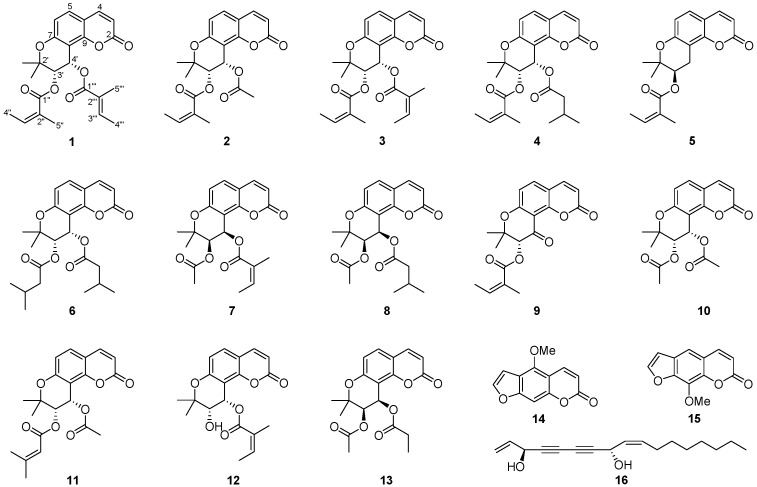
Structures of compounds **1**–**16** isolated from root extracts of *Peucedanum praeruptorum*.

The phytochemical analysis of the roots of *P. praeruptorum* using chromatographic separation methods resulted in the isolation of a new angular-type pyranocoumarin (**1**) as well as 12 pyranocoumarins (**2**–**13**), two furanocoumarins (**14**, **15**), and a polyacetylene (**16**). The structures of isolated compounds were elucidated by analyzing their spectroscopic data including NMR (1D and 2D) and high-resolution-electrospray ionization-mass spectrometry (HR-ESI-MS) as well as by comparing these data with reported values in the literature (Figure 1).

**Figure 2:**
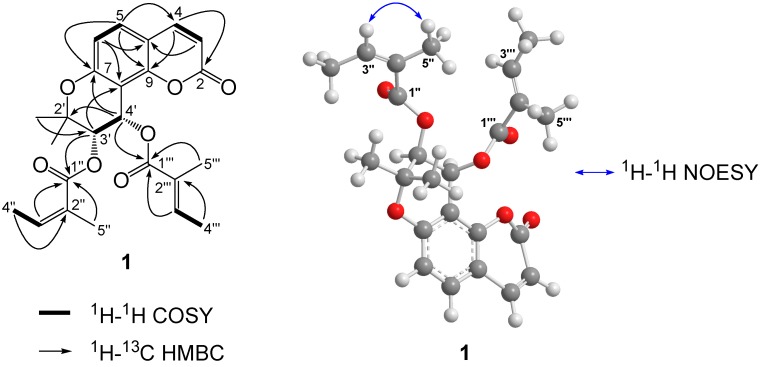
Key COSY, HMBC, and NOESY correlations of compound **1**. MM2 energy-minimized 3D structure acquired by a Chem3D Ultra software.

Compound **1** was obtained as a white powder with the molecular formula C_24_H_26_O_7_, deduced from the HR-ESI-MS peak at *m*/*z* 449.1572 [M + Na]^+^ (calcd. for C_24_H_26_O_7_Na, 449.1576). The ultraviolet (UV) spectrum showed maximal absorptions at 227 and 321 nm, indicating the presence of a coumarin moiety. Two pairs of doublet signals [δ 6.21 (1H, d, *J* = 9.5 Hz, H-3), 7.58 (1H, d, *J* = 9.5 Hz, H-4), 7.35 (1H, d, *J* = 8.7 Hz, H-5), 6.81 (1H, d, *J* = 8.7 Hz, H-6)] in the proton (^1^H)-NMR spectrum also supported the presence of a C-7 oxygenated coumarin moiety. The ^1^H-NMR spectrum showed two oxygenated methines [δ 5.44 (1H, d, *J* = 4.8 Hz, H-3′), 6.68 (1H, d, *J* = 4.8 Hz, H-4′)] with a characteristic splitting pattern and a germinal dimethyl group [δ 1.45 (3H, s, H-5′), 1.50 (3H, s, H-6′)] of a dihydropyran ring. The characteristic signals of an angeloyl group [δ 6.11 (1H, br q, *J* = 7.3 Hz, H-3′′), 1.94 (3H, dd, *J* = 1.2, 7.3 Hz, H-4′′), 1.82 (3H, m, H-5′′)] and a tigloyl group [*δ* 6.78 (1H, br q, *J* = 7.2 Hz, H-3′′′), 1.75 (3H, br d, *J* = 7.1 Hz, H-4′′′), 1.81 (3H, m, H-5′′′)] were observed from the ^1^H-NMR spectrum. The presence of these functional groups was also supported by the carbon (^13^C)-, distortionless enhancement by polarization transfer (DEPT), heteronuclear single quantum correlation (HSQC), and correlation spectroscopy (COSY) NMR spectra, suggested an angular-type pyranocoumarin khellactone diester. The connectivity between aromatic protons (H-5/H-6) and between two protons (H-3/H-4) of the α,β-unsaturated lactonic moiety were observed using ^1^H-^1^H COSY spectrum. The connectivity between two vicinal methine protons (H-3′/H-4′) was also observed in the ^1^H-^1^H COSY spectrum. Further, the correlation peaks between H-3′′/H-4′′ as well as H-3′′′/H-4′′′ were confirmed by the ^1^H-^1^H COSY spectrum (Figure 2). The positions of the two substituent groups were determined using the heteronuclear multiple-quantum correlation (HMBC) spectrum. The HMBC cross peaks of H-3′ with C-1′′ and H-4′ with C-1′′′ demonstrated that the angeloyl and tigloyl groups are connected to C-3′ and C-4′, respectively. The nuclear overhauser effect spectroscopy (NOESY) correlation between H-3′′ and H-5′′ was observed, whereas no NOESY correlation between H-3′′′ and H-5′′′ was observed, which also demonstrated the presence of a tigloyl group (Figure 2). The remaining positions of the quaternary carbons were also assigned based on the HMBC cross peaks (Figure 2).

From the spectral data obtained, compound **1** was found to be a new angular-type pyranocoumarin, 3′-angeloyl-4′-tigloylkhellactone, which was similar to (+)-praeruptorin B (**3**), (+)-*cis*-(3′*S*,4′*S*)-3′,4′-diangeloylkhellactone, except for the tigloyl group. The *cis* configuration between the two chiral centers, C-3′/C4′ was determined based on its large coupling constant *J*_3′4′_ as 4.8 Hz and large differences in chemical shifts (Δ = 2.6 ppm) between two germinal methyl signals in the ^13^C-NMR spectrum [6,18]. The absolute configuration was determined by comparing the optical rotation value (${\left[\alpha \right]}_{D}^{25}$ + 9.5, CHCl_3_) with those of some known analogues [19]. Therefore, compound **1** was established as (+)-*cis*-(3′*S*,4′*S*)-3′-angeloyl-4′-tigloylkhellactone.

The 15 known compounds were identified as (+)-praeruptorin A (**2**) [20,21], (+)-praeruptorin B (**3**) [20,21], (+)-praeruptorin E (**4**) [20,21,22], selinidin (**5**) [23], *cis*-3′,4′-diisovalerylkhellactone (**6**) [19,24], pteryxin (**7**) [6], suksdorfin (**8**) [25,26], Pd-Ib (**9**) [27,28], qianhucoumarin D (**10**) [20,21], (+)-samidin (**11**) [25,29,30,31], laserpitin (**12**) [32], (9*R*,10*R*)-9-acetoxy-8,8-dimethyl-9,10-dihydro-2*H*,8*H*-benzo[1,2-*b*:3,4-*b*′]dipyran-2-one-10-yl-ester (**13**) [33], bergapten (**14**) [34,35], xanthotoxin (**15**) [36], and falcalindiol (**16**) [37,38,39,40].

**Figure 3:**
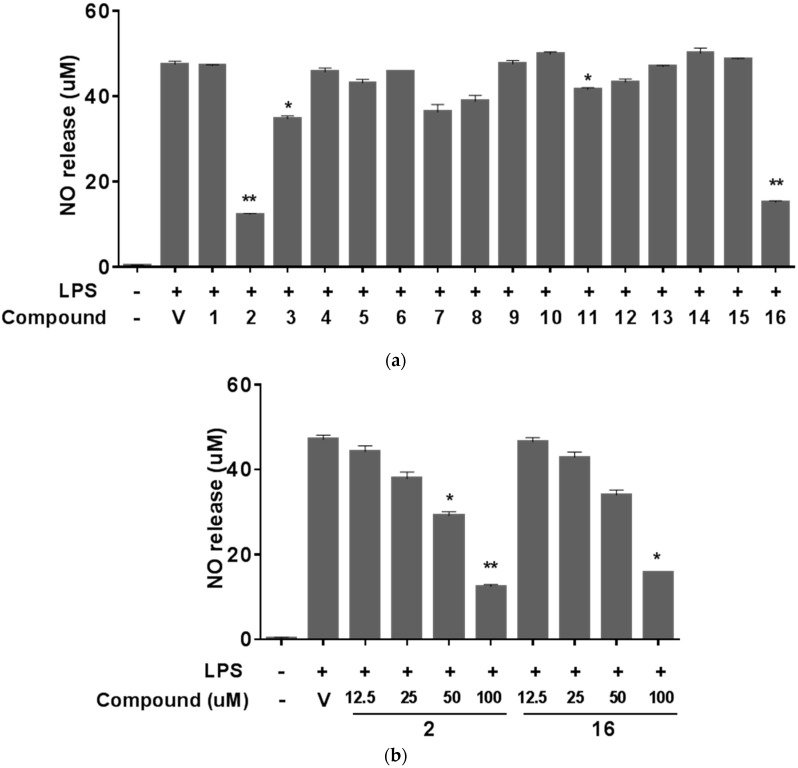
Effects of isolated compounds on nitric oxide production. Raw 264.7 cells were stimulated with 1 μg/mL LPS and co-treated with (**a**) 100 μM of each compound or vehicle (0.1% DMSO in PBS) as a control or (**b**) indicated compounds. Nitrite concentration in media was quantified using nitrite standard reference curve supplied in commercial Griess Reagent System. Data are means ± SD of one duplicated representative experiment. Differences between each treatment group against LPS control group were analyzed and statistical significances are denoted as * *p* < 0.05 or ** *p* < 0.01. LPS, lipopolysaccharide; DMSO, dimethyl sulfoxide; PBS, phosphate-buffered saline; SD, standard deviation.

**Figure 4:**
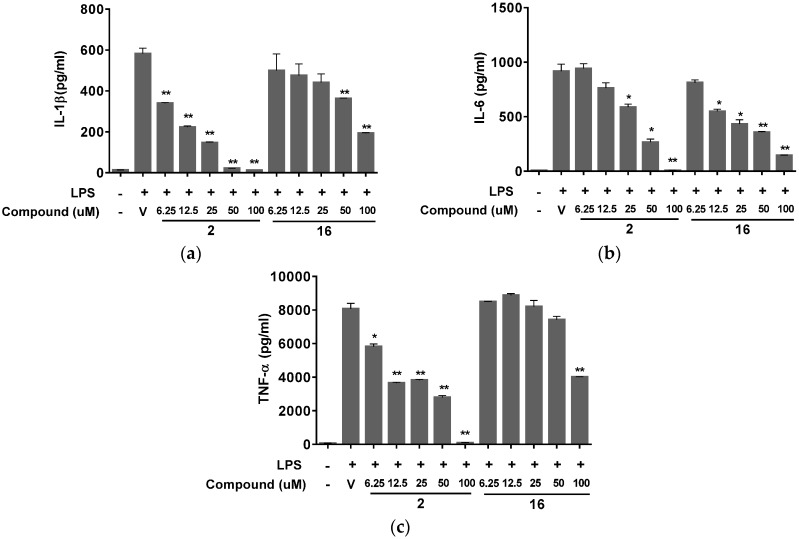
Effects of compound **2** and compound **16** on soluble mediators of inflammatory response. Raw 264.7 cells were stimulated with 1 μg/mL LPS and co-treated with various concentrations of compound **2**, compound **16** or vehicle (0.1% DMSO in PBS) as a control. After 24 h, the concentrations of IL-1β (**a**); IL-6 (**b**); and TNF-α (**c**) rerelease in the culture supernatants were determined using ELISA kit for each mediator. Data are means ± SD of one duplicated representative experiment. Differences between each treatment group against LPS control group were analyzed and statistical significances are denoted as * *p* < 0.05 or ** *p* < 0.01. LPS, lipopolysaccharide; DMSO, dimethyl sulfoxide; PBS, phosphate-buffered saline; SD, standard deviation.

*P. praeruptorum* roots and the constituents have been reported to have modulatory effects on tumor cells such as chemopreventive [14], anti-inflammatory [10,11,12], and multidrug resistance reversal [41,42]. Therefore, we examined the biological activities of the compounds isolated from the root extracts of *P. praeruptorum* using several *in vitro* assays to evaluate various aspects of their potential anticancer properties. First, the cytotoxic effects of the isolated compounds were investigated using A549 human non-small cell lung cancer cells, which were treated with varying concentrations of the test compounds at up to 100 μM for 48 h. Then, the cell viability was measured using a water-soluble tetrazolium salt (WST) assay (Ez-Cytox, Daeil Lab Service, Seoul, Korea). As expected, none of the compounds showed significant cytotoxicity or growth arrest in A549 lung cancer cells (data not shown). Next, we examined the effects of the isolated compounds on nitric oxide (NO) production in Raw 264.7 mouse macrophages stimulated with LPS. NO is mainly produced from l-arginine by the inducible nitric oxide synthase (iNOS) and is known to play a role in the host defense system against bacterial or viral infections or both by inducing inflammatory condition [43]. However, prolonged or hyper-stimulated NO production not only has the propensity to damage host cells but also contributes to cancer development by regulating the expression of genes involved in tumorigenesis [44,45,46]. Based on these scientific observations, NOS inhibitors and compounds that reduce the upregulation of NO production are considered as possible cancer chemotherapeutic candidate agents [43]. Of the compounds (**1**–**16**) isolated from the roots of *P. praeruptorum*, two of them (**2** and **16**) reduced the production of NO dose-dependently and by more than 70% at 100 μM in Raw 264.7 cells stimulated with 1 μg/mL LPS than the vehicle control did (Figure 3a,b). In addition to NO, pro-inflammatory cytokines such as tumor necrosis factor-α (TNF-α), interleukin-1β (IL-1β), and IL-6 are secreted from macrophages during inflammatory response and recognized as pivotal markers of inflammation [47,48]. Hence we further confirmed the anti-inflammatory effect of compound **2** and **16** on the secretion of these cytokines from the LPS-stimulated Raw 264.7 cells. Stimulation of cells with LPS markedly induced the release of IL-1β (Figure 4a), IL-6 (Figure 4b), and TNF-α (Figure 4c), which were suppressed by both compound **2** and compound **16** in a dose dependent manner, indicating that these compounds isolated from the roots of *P. praeruptorum* inhibit the early phase of LPS-stimuated inflammatory response.

**Figure 5:**
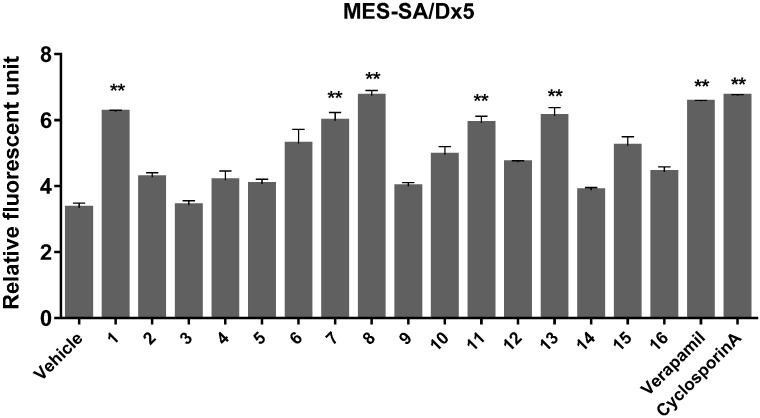
Inhibitory effects of isolated compounds against multidrug-resistant (MDR) protein-mediated drug efflux. MES-SA/Dx5 cells were treated with 10 μM of each compound and vehicle (0.1% DMSO in PBS), while verapamil or cyclosporine A were controls. This was followed by addition of cell-based Calcein AM/Hoechst dye staining solution, and its uptake was analyzed using a plate reader and normalized to cell densities measured using fluorescence intensity of Hoechst dye staining. Data are means ± SD of one duplicated representative experiment. Differences between each treatment group against LPS control group were analyzed and statistical significances are denoted as ** *p* < 0.01. DMSO, dimethyl sulfoxide; PBS, phosphate-buffered saline; SD, standard deviation.

It is a well-known fact that many drug-resistant tumor cells overexpress P-glycoprotein (Pgp), multidrug resistance-associated proteins (MRPs), or both, which decrease the cellular concentration of anticancer drugs and lead to MDR [41]. Furthermore, it has been reported that pyrocoumarins isolated from *P. praeruptorum* Dunn such as (±)-3′-angeloyl-4′-acetoxy-*cis*-khellactone (Pd-la), can suppress Pgp expression, reversing the MDR it induces, and consequently sensitize drug-resistant cancer cells to common anticancer agents [42]. In the present study, we used calcein-AM, a cell-permeable MDR protein substrate to test the MDR reversing activities of the compounds isolated from the roots of *P. praeruptorum* in the multidrug-resistant MES-SA/Dx5 cancer cell line. As shown in Figure 4, a few compounds showed enhanced calcein-AM fluorescence intensities, indicating a reduction in the drug-eliminating activities of MDR proteins. In particular, five compounds (**1**, **7**, **8**, **11**, and **13**) showed considerably significant activities compared to those of known MDR inhibitors (verapamil and cyclosporine A, Figure 5). Previous phytochemical investigations revealed that compounds **2** and **4** inhibited LPS-induced NO production in macrophages [12] and compounds **2**–**4** showed MDR reversal activities in cancer cells [42,46]. However, our study appears to be the first report of the anti-inflammatory potential of compound **16** and the potential MDR reversal activity of compounds **1**, **7**, **8**, **11**, and **13** in tumor cells.

## 3. Experimental Section

### 3.1. Materials and Major Equipment

Optical rotations were obtained using a P-2000 polarimeter (JASCO, Tokyo, Japan) and UV spectra were measured using an Ultrospec 8000 spectrophotometer (GE healthcare Life Science, Piscataway, NJ, USA). The HR-ESI-MS were obtained using a hybrid quadrupole orthogonal time-of-flight (Q-TOF) mass spectrometer (SYNAPT G2, Waters, MS Technologies, Manchester, UK) coupled with an ESI source. The NMR experiments were conducted on an Advance 500 FT-NMR (Bruker, Rheinstetten, Germany) with tetramethylsilane (TMS) as an internal standard. The thin layer chromatography (TLC) analysis was performed on silica gel 60 F_254_ and RP-18 F_254S_ plates (both Merck, Darmstadt, Germany). Silica gel (230–400 mesh, Merck, Darmstadt, Germany), reversed-phase silica gel (YMC, ODS-A, 12 nm, S-150 μm, Kyoto, Japan), Sephadex LH-20 (Sigma-Aldrich, St. Louis, MO, USA), and Cosmosil 140C_18_-OPN (Nacalai Tesque, Kyoto, Japan) were used for the chromatographic separation. Flash chromatography was performed using an Isolera One flash purification system (Biotage, Uppsala, Sweden). Pre-packed cartridges, a SNAP Ultra (340, 100, and 25 g) and a SNAP KP-C18-HS (120 and 30 g, Biotage) were used for flash chromatography. Dry load cartridges (100, 25, and 10 g scales, Biotage) manually packed with Sephadex LH-20 and LiChroprep RP-C18 (40–63 µm, Merck, Darmstadt, Germany) resins were also used for flash chromatography. Preparative (prep)-liquid chromatography (LC) was performed using an Agilent 1260 Infinity Preparative high-performance LC (HPLC) system (Agilent Technology, Waldbronn, Germany). A Prep-HPLC system consisting of a G1361A peristaltic pump, G1364B fraction collector, G1365D multiple wavelength detector, G2260A autosampler, semi-preparative columns, Chiralpak IB (5 µm, 250 × 10 mm i.d., Daicel Corporation, Tokyo, Japan), and Luna Silica (2) AXIA (5 µm, 250 mm × 10 mm i.d., Phenomenex, Torrance, CA, USA), were used for prep-HPLC. The system was operated using the OpenLAB CDS software (ChemStation Edition, Agilent Technologies, Santa Clara, CA, USA). HPLC grade acetonitrile (Baker, Center Valley, PA, USA) and ultrapure water (Millipore RiOs and Milli-Q-purification system, EMD Millipore, Billerica, MA, USA) were used for the isolation of the compounds.

### 3.2. Plant Material

The dried roots of *P. praeruptorum* were purchased from Kwangmyungdang Medicinal Herbs Co., (Ulsan, Korea) and identified by Dr. Go Ya Choi, K-herb Research Center, Korea Institute of Oriental Medicine, Korea. A voucher specimen (KIOM-CRC-50) was deposited at the KM Convergence Research Division, Korea Institute of Oriental Medicine, Korea.

### 3.3. Extraction and Isolation of Compounds

The plant material (10 kg) was ground and extracted thrice with 70% EtOH (40 L for 48 h each time) by maceration at room temperature. The extracts were filtered (Whatman filter paper, No. 2, Whatman International, Maidstone, UK), concentrated (EYELA rotary evaporation system, 20 L scale, 40 °C, Tokyo Rikakikai, Tokyo, Japan), and dried (WiseVen vacuum oven, WOW-70, Daihan Scientific, Seoul, Korea) to obtain the EtOH extract (2.0 kg). Then, 1.0 kg of the EtOH extract was suspended in distilled water and subsequently partitioned with organic solvents to obtain the *n*-hexane-, EtOAc-, *n*-BuOH-, and water-soluble extracts with yields of 118.7, 15.8, 77.3, and 786.2 g, respectively.

The *n*-hexane-soluble extract (118.7 g) was fractionated using a flash chromatography system with a SNAP Ultra cartridge (340 g, *n*-hexane:EtOAc, 95:5 to 50:50, CHCl_3_:acetone, 90:10 to 50:50, *v*/*v*) to obtain 29 subfractions (F01–F29). The F12 fraction (2.23 g) was further fractionated using a flash chromatography system with a SNAP KP-C18 cartridge (120 g, MeOH:water, 70:30, *v*/*v*) to obtain nine subfractions (F12-01–F12-09). Compound **5** (6.0 mg) was then separated from F12-03 (26.0 mg) using a flash chromatography system with a SNAP KP-C18 cartridge (30 g × 2, MeOH:water, 70:30, *v*/*v*). Subfraction F12-07 (1.06 g) was separated using a flash chromatography system with a SNAP KP-C18 cartridge (120 g, MeOH:water, 70:30, *v*/*v*) to obtain nine subfractions (F12-07-01–F14-07-09). Separation of compound **4** (76.5 mg) from F12-07-03 (644.1 mg) was performed using a flash chromatography system with a SNAP KP-C18 (120 g, MeOH:water, 65:35, *v*/*v*) and SNAP Ultra (100 g, *n*-hexane:EtOAc, 90:10 to 80:20) cartridges. Compound **6** (8.81 mg) was also separated from F12-07-03 using a flash chromatography (SNAP KP-C18, 120 g, MeOH:water, 65:35, *v*/*v*) and a prep-HPLC (Chiralpak IB semi-preparative column, 5 µm, 250 mm × 10 mm i.d., *n*-hexane:EtOAc, 95:5, *v*/*v*, flow rate 4 mL/min, UV 322 nm).

F14 (4.6 g) was subjected to flash chromatography using a SNAP KP-C18 cartridge (120 g, MeOH:water, 80:20, *v*/*v*) to obtain 11 subfractions (F14-01–F14-11). Chromatographic separation of F14-03 (276.5 mg) was also performed using a flash chromatography system with a SNAP Ultra (100 g, *n*-hexane:EtOAc, 80:20 to 70:30, *v*/*v*), SNAP KP-C18 (120 g, MeOH:water, 60:40 to 70:30, *v*/*v*), Sephadex LH-20 (100 g scale, MeOH:water, 70:30, *v*/*v*), Lichroprep RP-C18 (100 g scale, MeOH:water, 70:30, *v*/*v*), and SNAP Ultra (25 g × 2, CHCl_3_:MeOH:water, 19:1:0.05, *v*/*v*/*v*) cartridges to obtain compound **16** (8.48 mg).

F17 (3.95 g) was fractionated using a flash chromatography system using a SNAP KP-C18 cartridge (120 g, MeOH:water, 70:30 to 100:0, *v*/*v*) to obtain 19 subfractions (F17-01–F17-19). Subfractions F17-06 (255.2 mg) and F17-09 (700.3 mg) were fractionated using a flash chromatography system with a SNAP KP-C18 cartridge (120 g, MeOH:water, 60:40, *v*/*v*) to obtain compounds **7** and **8** (14.58 and 82.97 mg, respectively). Fractionation of F17-10 (135.2 mg) was performed using a flash chromatography system with a SNAP KP-C18 cartridge (120 g, MeOH:water, 60:40, *v*/*v*) to obtain nine subfractions (F17-10-01–F17-10-09). From F17-10-05 (66.13 mg), compound **1** (14.12 mg) was separated using a flash chromatography system with a SNAP Ultra cartridge (25 g × 2, *n*-hexane:EtOAc, 90:10 to 85:15, *v*/*v*).

F19 (10.51 g) was fractionated using a flash chromatography system with a SNAP KP-C18 cartridge (120 g, MeOH:water, 60:40, *v*/*v*) to produce 10 subfractions (F19-01–F19-10). Compound **14** (17.83 mg) was purified from F19-04 (37.78 mg) by crystallization. F20 (6.4 g) was subjected to flash chromatography using a SNAP KP-C18 cartridge (120 g, MeOH:water, 55:45 to 70:30, *v*/*v*) to obtain 11 subfractions (F20-01–F20-11). Compound **15** (16.90 mg) was obtained from F20-03 (58.24 mg) by crystallization.

Flash chromatography of F22 (791.2 mg) was carried out using a SNAP KP-C18 cartridge (120 g, MeOH:water, 60:40 to 70:30, *v*/*v*) to produce 13 subfractions (F22-01–F22-13). Fractionation of F22-07 (73.12 mg) was performed using a flash chromatography system with a SNAP KP-C18 cartridge (120 g, MeOH:water, 60:40, *v/v*) to produce four subfractions (F22-07-01–F22-07-04). Repeated flash chromatography of F22-07-02 (64.4 mg) was performed using a SNAP Ultra cartridge (25 g × 2, *n*-hexane:EtOAc, 90:10, *v/v*) and a SNAP KP-C18 cartridge (30 g × 2, MeOH:water, 55:45, *v/v*) and then, further purification of subfractions using a prep-HPLC [(Luna silica (2) semi-preparative column, 5 µm, 250 mm × 21.20 mm i.d., *n*-hexane:EtOAc, 70:30 (0–25 min) to 30:100 (25–50 min), *v/v*, flow rate 15 mL/min, UV 322 nm)] to produce compounds **12** (12.05 mg) and **13** (3.82 mg). Fractionation of F22-10 (233.22 mg) was performed using a flash chromatography system with a SNAP Ultra cartridge (100 g, *n*-hexane:EtOAc, 80:20 to 70:30, *v/v*) to produce five subfractions (F22-10-01–F22-10-05). Further chromatographic separation of F22-10-04 (106.96 mg) was performed using a flash chromatography system with a SNAP KP-C18 cartridge (120 g, MeOH:water, 60:40, *v/v*) and a SNAP Ultra cartridge (25 g × 2, *n*-hexane:EtOAc, 75:25, *v/v*) to produce compound **11** (10.34 mg).

**Scheme 1:**
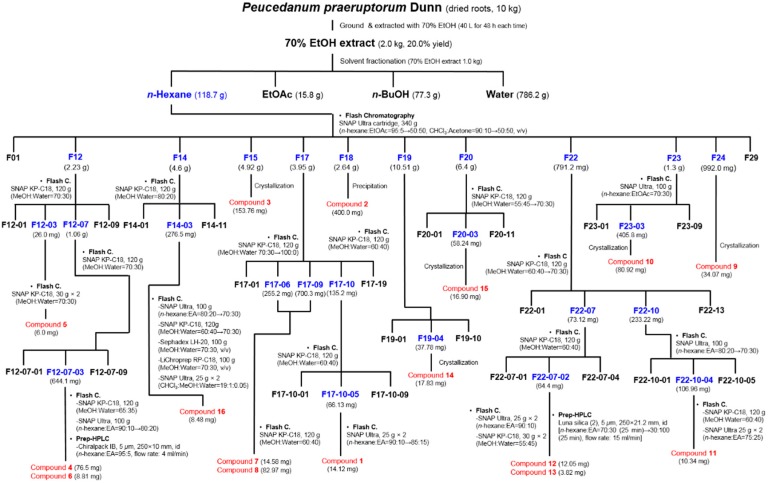
Extraction and isolation of compounds **1**–**16** from *P. praeruptorum.*

Fractionation of F23 (1.3 g) was carried out using a flash chromatography system with a SNAP Ultra cartridge (100 g, *n*-hexane:EtOAc, 70:30, *v/v*) to obtain nine subfractions (F23-01–F23-09). Compound **10** (80.92 mg) was obtained by crystallization from F23-03 (405.8 mg). Compounds **3** and **9** (153.76 and 34.07 mg, respectively) were also obtained by recrystallization from F15 and F24 (4.92 and 992.0 mg), respectively. Compound **2** (400.0 mg) was obtained from F18 (2.64 g) by precipitation. The procedure for the isolation of compounds **1**–**16** is shown in Scheme 1.

### 3.4. Characterization Data of (+)-cis-(3′S,4'S)-3'-Angeloyl-4′-tigloylkhellactone (***1***)

Compound **1** was obtained as a white powder with the following spectral characteristics ${\left[\alpha \right]}_{D}^{20}$ + 9.5 (*c* 0.1, CHCl_3_); UV (MeOH) *λ_max_* (log ε) 227 (4.22), 321 (4.12) nm; ^1^H-NMR (CDCl_3_, 500 MHz) δ 7.58 (1H, d, *J* = 9.5 Hz, H-4), 7.35 (1H, d, *J* = 8.7 Hz, H-5), 6.81 (1H, d, *J* = 8.7 Hz, H-6), 6.78 (1H, br q, *J* = 7.2 Hz, H-3′′′), 6.68 (1H, d, *J* = 4.8 Hz, H-4′), 6.21 (1H, d, *J* = 9.5 Hz, H-3), 6.11 (1H, br q, *J* = 7.3 Hz, H-3′′), 5.44 (1H, d, *J* = 4.8 Hz, H-3′), 1.94 (3H, dd, *J* = 1.2, 7.3 Hz, H-4′′), 1.82 (3H, m, overlapping, H-5′′), 1.81 (3H, m, overlapping, H-5′′′), 1.75 (3H, br d, *J* = 7.1 Hz, H-4′′′), 1.50 (3H, s, H-6′), 1.45 (3H, s, H-5′); ^13^C-NMR (CDCl_3_, 125 MHz) *δ* 166.9 (C-1′′′), 166.4 (C-1′′), 160.0 (C-2), 156.9 (C-7), 154.3 (C-9), 143.4 (C-4), 139.9 (C-3′′), 137.6 (C-3′′′), 129.3 (C-5), 128.5 (C-2′′′), 127.2 (C-2′′), 114.5 (C-6), 113.5 (C-3), 112.7 (C-10), 107.7 (C-8), 77.7 (C-2′), 70.3 (C-3′), 60.9 (C-4′), 25.5 (C-5′), 22.9 (C-6′), 20.6 (C-5′′), 15.9 (C-4′′), 14.6 (C-4′′′), 12.3 (C-5′′′); HRESIMS *m*/*z* 449.1572 [M + Na]+ (calcd for C_24_H_26_O_7_Na, 449.1576).

### 3.5. Cell Culture and Cell Viability

The MDR human uterine sarcoma MES-SA/Dx5 and RAW 264.7 mouse macrophage cell lines were cultured in McCoy’s 5A and Dulbecco’s modified Eagle’s medium (DMEM), respectively, each supplemented with 10% fetal bovine serum (FBS), 100 U/mL penicillin, and 100 μg/mL streptomycin (Invitrogen, Carlsbad, CA, USA), and maintained at 37 °C in a humidified incubator with 5% (*v*/*v*) CO_2_ atmosphere. All the cell lines used in this study were purchased from the American Type Culture Collection (ATCC, Manassas, VA, USA). Cell viability was quantified using the Ez-Cytox cell viability assay kit (Daeil Lab Service, Seoul, Korea) as previously described [49].

### 3.6. NO Assay

Raw 264.7 cells were inoculated at a density of 5 × 10^5^ cells/well in 48-well cell culture plates, cultured overnight, and then treated with 1 μg/mL LPS (Sigma-Aldrich) in the presence or absence of varying concentrations of the test compounds. After 20 h, the concentration of nitrite, a stable metabolite of NO, in the culture medium was measured using the Griess Reagent System (Promega, Madison WI, USA) following the manufacturer’s instructions.

### 3.7. Measurement of IL-1β, IL-6, and TNF-α

Raw 264.7 cells were inoculated at a density of 5 × 10^5^ cells/well in 48-well cell culture plates, cultured overnight, and then treated with 1 μg/mL LPS in the presence or absence of varying concentrations of the test compounds. After 24 h, culture supernatants were collected after centrifugation at 14,000 rpm for 10 min. Levels of IL-1β, IL-6, and TNF-α in the culture media from each group were determined by enzyme-linked immunosorbent assay (ELISA; R & D Systems, Minneapolis, MN, USA) per manufacturer’s instructions.

### 3.8. MDR Assay

MES-SA/Dx5 cells were seeded at a density of 5 × 10^4^ cells/well in 96-well plates containing 100 μL culture medium and grown overnight. Then, the cells were treated with 10 μM of the test compounds or vehicle control, as well as cyclosporine A or verapamil as the positive controls for 30 min at 37 °C in a humidified incubator with a 5% (*v*/*v*) CO_2_ atmosphere. Following the treatments, the MDR protein modulatory activities of the test compounds were measured using Calcein AM/Hoechst dye staining solution (Cayman Chemical, Ann Arbor, MI, USA) as described in the manufacturer’s instructions.

## 4. Conclusions

In the present study, a new angular-type pyranocoumarin, (+)-*cis*-(3′*S*,4′*S*)-3′-angeloyl-4′-tigloylkhellactone (**1**) was isolated from *P. praeruptorum* by chromatographic separation of a 70% EtOH extract of its roots. In addition, 15 known compounds (**2**–**16**) were also obtained. The structures of **1**–**16** were determined by spectroscopical data interpretation. The isolated compounds were subsequently evaluated for cytotoxic, MDR reversal, and anti-inflammatory activities against lung cancer, MES-SA/Dx5 cancer, and LPS-induced Raw 264.7 cells, respectively. Although none of the compounds exhibited cytotoxicity against the lung cancer cells (data not shown), five of them (**1**, **7**, **8**, **11**, and **13**) showed considerably significant MDR-reversal activity in the multidrug resistant MES-SA/Dx5 cancer cells. Furthermore, two compounds (**2** and **16**) showed anti-inflammatory activity by reducing the NO production and release of IL-1β, IL-6 and TNF-α induced by LPS stimulation of macrophage cells. Taken together, these data suggest that *P. praeruptorum* root and its constituents could be useful sources of candidates for the development of anticancer medicines specifically targeted at the restoration of the sensitivity to chemotherapeutic agents.
